# Galectin-3 impacts *Cryptococcus neoformans* infection through direct antifungal effects

**DOI:** 10.1038/s41467-017-02126-7

**Published:** 2017-12-06

**Authors:** Fausto Almeida, Julie M. Wolf, Thiago Aparecido da Silva, Carlos M. DeLeon-Rodriguez, Caroline Patini Rezende, André Moreira Pessoni, Fabrício Freitas Fernandes, Rafael Silva-Rocha, Roberto Martinez, Marcio L. Rodrigues, Maria Cristina Roque-Barreira, Arturo Casadevall

**Affiliations:** 1 Department of Biochemistry and Immunology, Ribeirao Preto Medical School, University of Sao Paulo, Ribeirao Preto, SP 14049-900 Brazil; 2 Department of Microbiology and Immunology, Albert Einstein College of Medicine, Yeshiva University, New York, NY 10461 USA; 3 Department of Cellular and Molecular Biology, Ribeirao Preto Medical School, University of Sao Paulo, Ribeirao Preto, SP 14049-900 Brazil; 4 Department of Internal Medicine, Ribeirao Preto Medical School, University of Sao Paulo, Ribeirao Preto, SP 14048-900 Brazil; 5 Instituto de Microbiologia Paulo de Goes,, Universidade Federal do Rio de Janeiro, Rio de Janeiro, 21941-902 Brazil; 6 Fundação Oswaldo Cruz—Fiocruz, Centro de Desenvolvimento Tecnológico em Saúde (CDTS), Rio de Janeiro, 21041-361 Brazil; 7 Department of Molecular Microbiology and Immunology, Johns Hopkins Bloomberg School of Public Health, Baltimore, MD 21205 USA

## Abstract

*Cryptococcus neoformans* is an encapsulated fungal pathogen that causes cryptococcosis, which is a major opportunistic infection in immunosuppressed individuals. Mammalian β-galactoside-binding protein Galectin-3 (Gal-3) modulates the host innate and adaptive immunity, and plays significant roles during microbial infections including some fungal diseases. Here we show that this protein plays a role also in *C*. *neoformans* infection. We find augmented Gal-3 serum levels in human and experimental infections, as well as in spleen, lung, and brain tissues of infected mice. Gal-3-deficient mice are more susceptible to cryptococcosis than WT animals, as demonstrated by the higher fungal burden and lower animal survival. In vitro experiments show that Gal-3 inhibits fungal growth and exerts a direct lytic effect on *C*. *neoformans* extracellular vesicles (EVs). Our results indicate a direct role for Gal-3 in antifungal immunity whereby this molecule affects the outcome of *C*. *neoformans* infection by inhibiting fungal growth and reducing EV stability, which in turn could benefit the host.

## Abstract (web summary)

The protein Galectin-3 modulates host immunity and plays roles during infections. Here, Almeida et al. show that this protein contributes to host defence against infection with the fungal pathogen *Cryptococcus neoformans* by inhibiting fungal growth and inducing lysis of fungal extracellular vesicles.

## Introduction

Cryptococcosis, a disease that is mainly caused by *Cryptococcus neoformans*, is a major infection in immunocompromised hosts, such as those with advanced HIV infection^1^. *C*. *neoformans* is found worldwide in various environmental niches, generally associated with avian guano or vegetation^2, 3^. Exposure to *C*. *neoformans* usually does not cause overt disease and the infection may lead to an asymptomatic latent state^4, 5^. The reactivation of infection causes pneumonia and/or meningoencephalitis, which are frequently fatal—even when treated aggressively with antifungal drug therapy^6^. The most important virulence factors of *C*. *neoformans* are the polysaccharide capsule^7^, cell wall-associated melanin^8^, capacity to grow at body temperature^9^, and ability to produce extracellular enzymes^10^. These factors together with the host state can determine the outcome of the infection.

For all pathogenic microbes, extracellular release of molecules is a vital process^11^. A number of the important mechanisms through which fungal pathogens export molecules require trans-cell wall transport in extracellular vesicles (EVs)^11^. EVs carry several virulence factors and may contribute with fungal virulence and modulation of host immunity^12–15^. Vesicular stability is assumed to be important to ensure proper delivery of their content into host tissues and cells^16^.

Several lectins play immunomodulatory activities, mostly through interactions between their carbohydrate recognition domains (CRD)s with the glycan moieties of receptors of immune cells. Otherwise, host lectins may interact with sugars present or released by microbial cells, and play critical roles in infections. Galectin-3 (Gal-3), an animal lectin that typically binds β-galactosides, is a pleiotropic protein intimately involved in a variety of cellular processes. Extracellular Gal-3 can modulate adhesion, activation, and cellular migration, whereas intracellular Gal-3 regulates fundamental processes such as pre-mRNA splicing and phagocytosis^17^. Gal-3 plays important roles in the development and regulation of immunity homeostasis. During infections, Gal-3 exerts pro-inflammatory activity, enhances macrophages survival, and induces macrophage recruitment, antimicrobial activities, and cytokines production. Gal-3 is found deposited in pyogranuloma and granuloma in *Rhodococcus equi* ^18^ and *Schistosoma mansoni* ^19^ infections, respectively. During the infection caused by *S*. *mansoni*, Gal-3 binds to GalNAc β1,4GlcNAc-containing glycans, which constitute the most common *N*-linked glycan component in invertebrates. Binding of Gal-3 to *S*. *mansoni* results in increased phagocytosis by macrophages^19^. In addition to its immunomodulatory roles, Gal-3 can bind to glycans on the surface of pathogens, enabling or impeding microbial invasion, supporting their survival or leading to an effective host immune response^20^. Infecting microbes, in turn, can modulate Gal-3 expression, which regulates leukocyte functions and inflammatory responses. Consequently, Gal-3 significantly influences a number of microbial infections, a knowledge that is mostly derived from comparative studies on the course of models in Gal-3KO and WT mice^17, 18, 21–23^.

Mycoses whose course is influenced by Gal-3 include those caused by *Paracoccidioides brasiliensis* ^23^, *Candida albicans* ^24^, and *Histoplasma capsulatum* ^25^. Nevertheless there are no studies focusing on the role played by Gal-3 in cryptococcosis. In this study, we compared the severity of the *C*. *neoformans* infection in Gal-3KO and WT mice, assessed the Gal-3 content in organs of infected mice, and determined the Gal-3 serum levels in both experimental and human cryptococcosis. We also evaluated whether Gal-3 influenced *C*. *neoformans* growth and stability of EVs. Our results demonstrate that Gal-3 plays relevant roles in *C*. *neoformans* infection primarily through direct effects on cryptococcal cells and their products.

## Results

### Gal-3 is upregulated during *C*. *neoformans* infection

**Fig. 1:**
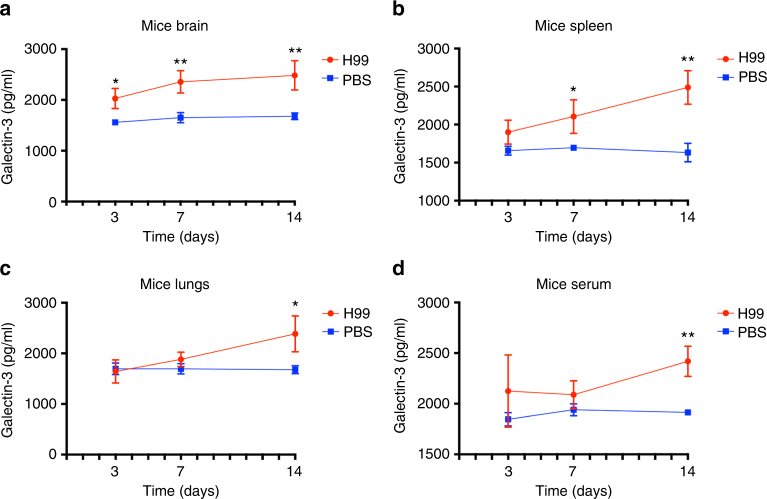
Upregulated Gal-3 levels in mice during experimental *C*. *neoformans* infection. C57BL/6 mice were intratracheally infected with H99 yeast cells (red line with circle) or PBS (blue line with square) and Gal-3 levels were verified in tissues and serum during the course of *C*. *neoformans* infection. On days 3, 7, and 14 after the fungal inoculation, samples collected of brain (**a**), spleen (**b**), lungs (**c**), and serum (**d**), were homogenized and assessed by ELISA regarding the Gal-3 concentration. Gal-3 levels were upregulated over time after infection with *C*. *neoformans*. Bars represent the mean ± SD of Gal-3 levels obtained from triplicate samples in groups of five animals. Statistically significant differences are denoted by asterisks (*p < 0.05, **p < 0.005, unpaired Student’s *t*-test)

Since augmented Gal3 expression was previously reported during human and experimental inflammatory diseases^26, 27^, we determined Gal-3 levels in tissues and serum of C57BL/6 mice on days 3, 7, and 14 post-infection with *C*. *neoformans*. In comparison with control animals, infected mice had higher Gal-3 levels in all examined tissues and serum samples (Fig. 1). In brain samples (Fig. 1a), Gal-3 levels were increased in mice on days 3, 7, and 14 post-infection. Similar results were observed in spleen samples (Fig. 1b), although statistical differences between control and infected animals were observed only in days 7 and 14 post-infection. In lung and serum samples (Fig. 1c, d, respectively), Gal-3 levels were higher only at day 14 post-infection. We concluded that the *C*. *neoformans* infection in mice led to increases in tissue and serum Gal-3 content, with a variable time-course depending on the tissue.

**Fig. 2:**
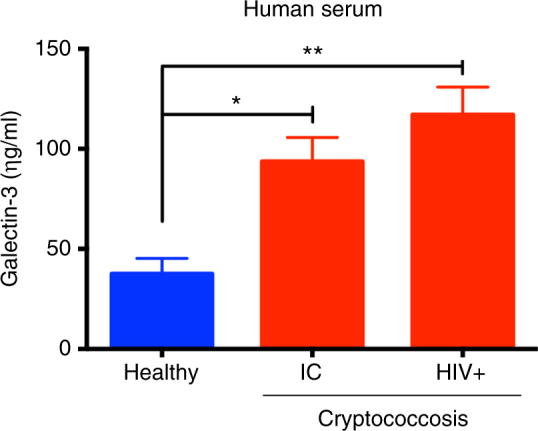
Upregulated Gal-3 levels in humans during *C*. *neoformans* infection. Gal-3 levels in serum from healthy individuals (blue bar) and patients infected by *C*. *neoformans* (red bars) were assessed by ELISA. Gal-3 levels were higher in IC (immunocompetent) and HIV (human immunodeficiency virus) patients infected with *C*. *neoformans* when compared with healthy individuals. Bars represent the mean ± SD of Gal-3 levels obtained from triplicate samples. Statistically significant differences are denoted by asterisks (*p < 0.05, **p < 0.005, unpaired Student’s *t*-test)

We then measured Gal-3 levels in serum samples from individuals suffering of cryptococcosis, belonging to groups of patients who were immunocompetent (IC) or HIV-positive. An additional control group, constituted by healthy individuals, was assessed for the Gal-3 serum levels. Compared to the healthy individuals, the IC and HIV+ patients showed higher Gal-3 serum levels, possibly attributed to the concurrent cryptococcosis (Fig. 2). There was no significant difference (*p*-value: 0.0926, *t*-test) between the Gal-3 levels in sera of IC and HIV+ patients with cryptococcosis. These results reinforce the notion, derived from the experimental studies, that there is a relation between *C*. *neoformans* infection and increased levels of serum Gal-3.

### Gal-3 contributes to the control of the *C*. *neoformans* infection

**Fig. 3:**
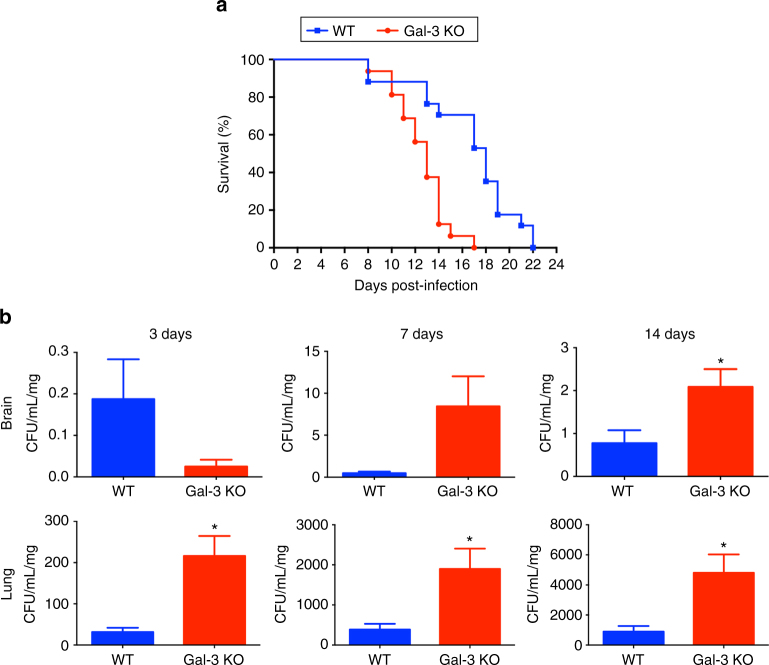
Absence of Gal-3 leads to lower survival and increased fungal burden in experimental murine cryptococcosis. The survival rate (**a**) of Gal-3 KO mice (red line with circle) and WT mice (blue line with square) were verified after intratracheally infection with H99 yeast cells. **b** Colony-forming units (CFU) recovered from brain and lungs of WT (blue bars) and Gal-3 KO (red bars) mice were assessed after 3, 7, and 14 days after intratracheally infected with *C*. *neoformans*. The *y*-axis denotes CFU/ml/mg resulting from normalization of fungal burden to the weight of the organ fragment used to prepare the homogenate. Data are representative of three experiments, each performed with eight mice per group. The bars represent the mean ± SD of CFU obtained from triplicate samples in groups of eight animals. *p < 0.05, Student’s *t*-test, Gal-3 KO mice compared with WT mice

To investigate whether the Gal-3 upregulation could influence the host resistance to the *C*. *neoformans* infection, we compared survival levels and fungal burden of Gal-3-deficient (gal3^−/−^) and WT mice. Gal-3-deficient mice died faster than WT animals (Fig. 3a). These results were supported by quantification of fungal burden in organs of both groups of mice. We recovered higher colony-forming units (CFU) from the lungs harvested at 3, 7, and 14 days post-infection from gal3^−/−^ mice than from WT mice (Fig. 3a). Regarding the brain, we recovered higher CFU number from gal3^−/−^ than from WT mice at 14 days post-infection (Fig. 3b). The set of experimental observations reported in this section reveals that Gal-3 is implicated in the control of the *C*. *neoformans* infection.

### Gal-3 deficiency affects TH17 immune response

**Fig. 4:**
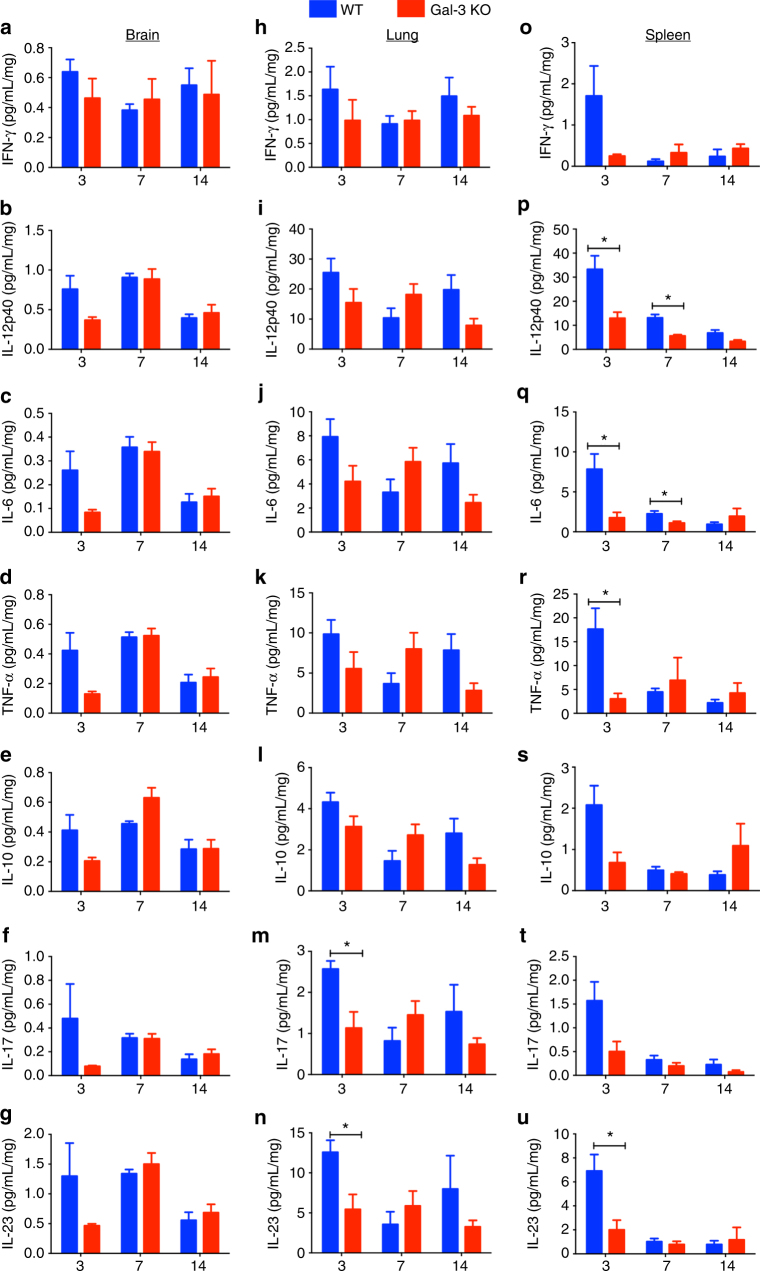
Levels of relevant cytokines in the brain, lung, and spleen of *C*. *neoformans*-infected WT and Gal-3 KO mice. WT (blue bars) and Gal-3 KO (red bars) mice were intratracheally infected with a 50 μl suspension containing 1×10^6^ yeast cells. Organ samples were weighed and homogenized. The levels of the following cytokines in the brains (**a**–**g**), lungs (**h**–**n**), and spleens (**o**–**u**) (3, 7, and 14 days post-infection) of infected mice were determined by ELISA: IFN-γ, IL-12p40, IL-6, TNF-α, IL-10, IL-17, and IL-23. The results represent the mean ± SD of five mice per group, from a representative experiment of three assays. *p < 0.05, Student’s *t*-test, Gal-3 KO mice compared with WT mice

**Fig. 5:**
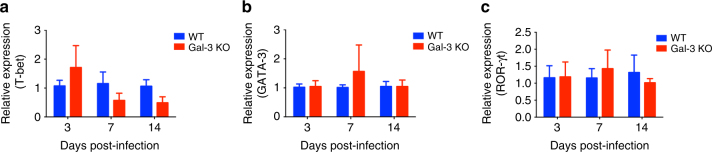
Levels of T-bet, GATA-3, and ROR-γt in the lungs of *C*. *neoformans*-infected WT and Gal-3 KO mice. WT (blue bars) and Gal-3 KO (red bars) mice were intratracheally infected with H99 yeast cells and samples of their lungs weighed and homogeneized. The levels of mRNA relative expression for T-bet (**a**), GATA-3 (**b**), and ROR-γt (**c**) in the lungs (3, 7, and 14 days post-infection) of infected mice were determined by real-time PCR, using the β-actin gene as control. The results represent the mean ± SD of five mice per group, from a representative experiment of three assays

We investigated whether the Gal-3 absence or presence drive distinctly the immune response toward the Th1, Th2 or Th17 axis during a *C*. *neoformans* experimental infection. To do so we assessed the cytokine content in the brain, lungs, and spleen, which were harvested at 3, 7, and 14 days post-infection from WT and gal3^−/−^ mice. The IFN-γ, IL-12p40, IL-6, TNF-α, IL-10 contents in the brain and lung homogenates did not differ between WT and gal3^−/−^ groups of mice, in all infection period (Fig. 4a–e, h–l, respectively). In contrast, the concentration of IL-12p40, IL-6, and TNF-α in the spleen homogenates from WT mice were higher than in gal3^−/−^ mice (Fig. 4p–r), whereas the spleen contents of IFN-γ and IL-10 were similar between the two groups of mice (Fig. 4c, s). Surprisingly, IL-17 and IL-23 contents in the lungs and spleen were higher than in gal3^−/−^ mice (Fig. 4m, n, t, u). We also evaluated the frequency of γδ T cells in pulmonary tissue at 3 days post-infection, which revealed that γδ T cells were more frequently detected in WT animals, in comparison to gal3^−/−^ mice (Supplementary Figure 1). This observation was in agreement with IL-17 detection in infected lungs. In addition, we evaluated the relative expression of transcription factors involved in T-cell differentiation, namely T-bet (for Th1 cells), GATA-3 (for Th2 cells), and ROR-γt (for Th17 cells) and found that the three transcription factors at days 3, 7, and 14 post-infection were similarly expressed in WT and gal3^−/−^ mice (Fig. 5). These results suggest that the Gal-3 role in the control of the *C*. *neoformans* infection promotes skewed TH17 immune response profile.

### Gal-3 inhibits the fungal growth

**Fig. 6:**
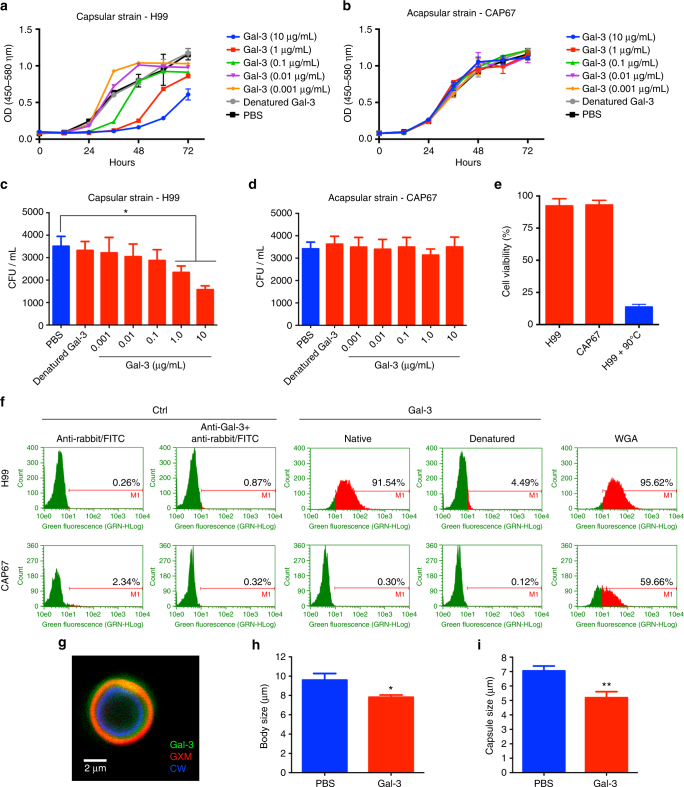
Gal-3 interacts with the *C*. *neoformans* capsule and inhibits the fungal growth. Capsular strain H99 (**a** and **c**) and acapsular strain CAP67 (**b** and **d**) were incubated for 72 h at 37 °C with Gal-3 (0.001 to 10 μg/ml), and the optical density at 540 nm (OD540) measured every hour (**a** and **b**), or the number or colony-forming units (CFU) were counted for determination of cell densities (**c** and **d**). **e** H99 and CAP67 strains were incubated for 72 h at 37 °C with Gal-3 (10 μg/ml), and the frequency of viable cells assessed using propidium iodide staining. Heated fungal cells (90 °C for 10 min) were used as positive control (blue bar). H99 and CAP67 strains were incubated for 40 min at 4 °C with Gal-3 40 μg/ml. After that, both strains were washed with PBS and incubated for 45 min with anti-Gal-3 antibody. Then, washed again with PBS and incubated with anti-rabbit IgG-FITC antibody for 40 min at 4 °C. Labeled cells were acquired on a FACS Guava easyCyte, and the histogram represents the percentage of positive cells recognized by Gal-3 (**f**). Anti-rabbit IgG-FITC antibody associated or not with Gal-3 was used as control, and use of WGA lectin (30 μg/ml) as control of binding with capsule or cell wall (**f**). **g** H99 strain was cultured at 37 °C for 24 h and incubated with Gal-3. *C*. *neoformans* were stained for observation of cell wall (CW) with calcofluor white (blue), Gal-3 with anti-Gal-3 antibody (green), and capsule with anti-GXM (red). Body (**h**), and capsule (**i**) size of H99 strain cells were assessed by microscopic analysis after cultivation in the presence of Gal-3 10 μg/ml. Data are representative of three experiments. Statistically significant differences are denoted by asterisk (*p < 0.05, unpaired Student’s *t*-test)

Since the role of Gal-3 in the experimental *C*. *neoformans* infection was not clearly related to an adaptive immune response, we investigated whether Gal-3 could directly recognize fungal surface sugars and consequently affect fungal cell physiology. Gal-3 promoted a dose-dependent delay in the growth of encapsulated cells of *C*. *neoformans* (Fig. 6a). Gal-3 did not affect the proliferation of a *C*. *neoformans* acapsular strain (CAP67, Fig. 6b). Similar results were obtained by CFU determination (Fig. 6c, d) and propidium iodide staining (Fig. 6e). Flow cytometry assessment of the Gal-3 binding to H99 and CAP67 *C*. *neoformans* cells showed that Gal-3 bound to the capsular H99 cells and not to the acapsular CAP67 cells. Denatured Gal-3 did not bind to the yeasts surface (Fig. 6f). Wheat germ agglutinin (WGA), used as a positive control, bound to yeasts of both strains (Fig. 6f). Confocal microscopy demonstrated that Gal-3 co-localized with the cryptococcal capsule (Fig. 6g and Supplementary Figure 2). These results suggest that the recognition of the fungal capsule by Gal-3 may account for its effect of inhibiting the in vitro *C*. *neoformans* growth.

We also studied the effect of Gal-3 on the cell body and capsule dimensions of *C*. *neoformans* after 72 h in the presence of Gal-3 (10 μg/ml). Compared to the control culture (phosphate-buffered saline (PBS)), the yeasts grown in the Gal-3 presence had reduced sizes of cell body and capsule (Fig. 6h, i, respectively).

To investigate the potential mechanisms involved in the Gal-3-mediated inhibition of *C*. *neoformans* growth, RNA-seq analysis was performed after the fungus was exposed to Gal-3. This analysis demonstrated Gal-3 exposure resulted in upregulation of 954 genes and downregulation of 1149 genes (Supplementary Data 1–4). Within the upregulated genes, 420 had annotated functions and 534 were hypothetical, while for the downregulated genes 750 were known and 399 were hypothetical. The 40 most affected upregulated and downregulated genes are listed in Tables 1 and 2, respectively. In these groups, upregulated genes were mostly related to membrane proteins, with a clear association with sugar transport. Downregulated genes were mainly related to proteins of the large and small ribosome subunits. Gene ontology (GO) analysis of repressed genes showed enrichment in genes related to ribosomes, ATP synthesis, mitochondria, and integral components of the plasma membrane, suggesting a general interference with energy metabolism. On the other hand, GO analysis of upregulated genes showed enrichment in genes related to protein kinases, dioxygenase, hydrolases, transmembrane transport, and intracellular processes.

**Table 1:** List of top 40 induced genes with annotated functions upon exposure to Gal-3

Gene	logFC	*p*-value	RPKM control	RPKM Gal-3	Gene ID	Annotation
XM_012192153.1	6.03	0.0000	0.8	55.2	CNAG_03910	D-xylose-proton symporter
XM_012194171.1	5.91	0.0000	0.0	2.4	CNAG_06812	Phospholipase D, variant
XM_012198528.1	5.45	0.0000	11.7	513.7	CNAG_05662	Sugar transporter
XM_012191199.1	4.87	0.0000	4.8	142.9	CNAG_00183	Alternative cyclin Pcl12, variant
XM_012191495.1	4.66	0.0407	1.3	33.0	CNAG_00680	Kinetochore protein Nuf2, variant
XM_012191826.1	4.39	0.0000	3.9	81.8	CNAG_03772	Glucose transporter
XM_012191299.1	4.17	0.0000	6.9	126.2	CNAG_00393	1,4-alpha-glucan-branching enzyme
XM_012198536.1	3.82	0.0000	1.2	16.8	CNAG_07874	Sugar transporter
XM_012196714.1	3.81	0.0000	12.0	170.1	CNAG_04621	Glycogen(starch) synthase, variant
XM_012192450.1	3.58	0.0000	31.8	384.2	CNAG_02943	Cytoplasmic protein
XM_012194439.1	3.48	0.0000	12.4	140.0	CNAG_02165	Cytoplasmic protein
XM_012193764.1	3.45	0.0000	2.7	30.0	CNAG_00864	MFS transporter, SP family, solute carrier family 2 (myo-inositol transporter), member 13
XM_012194787.1	3.44	0.0000	0.1	1.4	CNAG_02489	Alcohol dehydrogenase, propanol-preferring
XM_012194657.1	3.20	0.0000	6.1	56.7	CNAG_02269	Autophagy-like protein 18 Atg18
XM_012191442.1	3.12	0.0000	2.4	21.4	CNAG_00598	Nicotinamide mononucleotide permease
XM_012198344.1	2.87	0.0000	281.5	2069.0	CNAG_05387	Galactose transporter
XM_012191781.1	2.84	0.0000	3.5	25.2	CNAG_03666	Acyl-CoA dehydrogenase
XM_012193765.1	2.82	0.0001	0.5	3.3	CNAG_00865	Maltose *O*-acetyltransferase partial
XM_012192132.1	2.80	0.0000	8.7	61.1	CNAG_03876	Ras family protein
XM_012194860.1	2.74	0.0000	1.3	9.1	CNAG_07641	Monosaccharide transporter
XM_012194944.1	2.73	0.0000	106.2	709.0	CNAG_05685	Neutral amino acid transporter
XM_012194030.1	2.72	0.0000	5.6	37.0	CNAG_01252	Thiosulfate/3-mercaptopyruvate sulfurtransferase
XM_012191185.1	2.72	0.0000	1089.1	7234.7	CNAG_00162	Alternative oxidase, mitochondrial
XM_012192590.1	2.72	0.0000	1.8	12.3	CNAG_02592	Thioredoxin reductase GliT
XM_012197279.1	2.69	0.0000	10.3	66.7	CNAG_01653	Cytokine inducing-glycoprotein
XM_012191929.1	2.59	0.0000	9.7	58.6	CNAG_04070	Exonuclease
XM_012193425.1	2.53	0.0000	1.9	11.0	CNAG_05266	Membrane protein, variant
XM_012195873.1	2.51	0.0000	1.4	8.2	CNAG_03442	Phosphatidylinositol glycan, class T
XM_012195613.1	2.47	0.0000	4.3	24.2	CNAG_03563	Aspartate-tRNA(Asn) ligase
XM_012194884.1	2.47	0.0002	0.2	1.1	CNAG_06561	Allantoate transporter
XM_012193248.1	2.46	0.0000	45.7	254.0	CNAG_04981	Catalase
XM_012191909.1	2.44	0.0000	16.3	89.7	CNAG_03991	Integral membrane protein
XM_012193748.1	2.43	0.0000	5.3	28.9	CNAG_00883	Transcription factor
XM_012192625.1	2.42	0.0000	1.3	7.0	CNAG_06932	Sugar transporter
XM_012193344.1	2.39	0.0000	4.8	25.5	CNAG_05139	Solute carrier family 35 (UDP-sugar transporter), member A1/2/3, variant
XM_012194484.1	2.36	0.0000	9.7	50.1	CNAG_02028	CMGC/SRPK protein kinase
XM_012197123.1	2.33	0.0000	24.0	121.8	CNAG_01955	Alcohol dehydrogenase
XM_012194776.1	2.29	0.0000	5.3	26.3	CNAG_02475	Flavin-containing monooxygenase
XM_012197151.1	2.26	0.0000	432.1	2089.6	CNAG_01464	Nitric oxide dioxygenase
XM_012197669.1	2.24	0.0000	23.3	111.0	CNAG_06241	Acidic laccase

**Table 2:** List of top 40 repressed genes with annotated functions upon exposure to Gal-3

Gene	logFC	*p*-value	RPKM Control	RPKM Gal-3	Gene ID	Annotation
XM_012196089.1	−9.75	0.0000	2.2	0.0	CNAG_04297	Ribonuclease HII
XM_012192266.1	−4.10	0.0000	71.3	4.2	CNAG_04068	Large subunit ribosomal protein L28e
XM_012196872.1	−3.91	0.0117	3.4	0.2	CNAG_04868	Cytoplasmic protein, variant
XM_012197773.1	−3.90	0.0000	328.5	22.1	CNAG_06144	Cytoplasmic protein
XM_012192059.1	−3.82	0.0273	19.0	1.4	CNAG_03779	Large subunit ribosomal protein L4, variant
XM_012198492.1	−3.79	0.0099	1.7	0.1	CNAG_05617	Phosphatidylinositol glycan, class O, variant
XM_012191041.1	−3.77	0.0000	24.2	1.8	CNAG_00788	NADH dehydrogenase
XM_012192763.1	−3.70	0.0001	8.3	0.6	CNAG_02818	Glycine cleavage system T protein
XM_012193962.1	−3.65	0.0000	350.9	28.2	CNAG_01153	Small subunit ribosomal protein S13e
XM_012197430.1	−3.63	0.0000	26.4	2.1	CNAG_01877	GMP synthase [glutamine-hydrolyzing]
XM_012197752.1	−3.55	0.0000	47.8	4.1	CNAG_06112	Carbamoyl-phosphate synthase arginine-specific large chain
XM_012191489.1	−3.45	0.0000	166.5	15.4	CNAG_00672	Small subunit ribosomal protein S11
XM_012191559.1	−3.41	0.0000	67.1	6.4	CNAG_00771	Large subunit ribosomal protein L29
XM_012194664.1	−3.40	0.0000	94.9	9.0	CNAG_02285	Nucleoside diphosphate kinase
XM_012198108.1	−3.38	0.0000	183.1	17.8	CNAG_06447	Large subunit ribosomal protein L22
XM_012195922.1	−3.37	0.0000	54.3	5.3	CNAG_03519	Cytoplasmic protein, variant 1
XM_012197163.1	−3.36	0.0000	136.6	13.4	CNAG_01486	Large subunit ribosomal protein L15-A
XM_012192913.1	−3.33	0.0000	114.1	11.4	CNAG_03053	Large subunit ribosomal protein L23
XM_012196826.1	−3.32	0.0000	83.6	8.4	CNAG_04799	Large subunit ribosomal protein L14e
XM_012192296.1	−3.24	0.0000	27.4	2.9	CNAG_06717	Acyl-CoA-dependent ceramide synthase
XM_012194746.1	−3.23	0.0000	64.4	6.9	CNAG_02418	Asparagine-tRNA ligase
XM_012197503.1	−3.20	0.0000	123.9	13.6	CNAG_01990	Small subunit ribosomal protein S5
XM_012191565.1	−3.20	0.0000	99.6	10.9	CNAG_00779	Large subunit ribosomal protein L27e
XM_012198016.1	−3.17	0.0000	26.6	3.0	CNAG_06314	Phosphoribosylamine-glycine ligase
XM_012195288.1	−3.16	0.0006	2.1	0.2	CNAG_06539	Monocarboxylic acid transporter, variant
XM_012195161.1	−3.15	0.0000	208.1	23.5	CNAG_05814	Small subunit ribosomal protein S10e
XM_012191082.1	−3.15	0.0000	12.2	1.4	CNAG_00026	Kynurenine aminotransferase, variant
XM_012194011.1	−3.14	0.0000	114.2	13.0	CNAG_01224	Large subunit ribosomal protein L18-A
XM_012192836.1	−3.12	0.0000	90.3	10.5	CNAG_02928	Large subunit ribosomal protein L5e
XM_012195803.1	−3.11	0.0000	38.2	4.5	CNAG_03342	tRNA (guanine-N(7)-)-methyltransferase subunit TRM82
XM_012192217.1	−3.11	0.0000	203.4	23.7	CNAG_04004	Small subunit ribosomal protein S1
XM_012197477.1	−3.11	0.0000	162.8	19.0	CNAG_01951	Small subunit ribosomal protein S22-A
XM_012194723.1	−3.09	0.0000	56.7	6.7	CNAG_02378	H/ACA ribonucleoprotein complex subunit 2
XM_012196828.1	−3.08	0.0000	7.9	0.9	CNAG_04802	Ribosomal RNA-processing protein 9
XM_012196775.1	−3.05	0.0000	263.3	32.1	CNAG_04726	Large subunit ribosomal protein L18Ae
XM_012197836.1	−3.04	0.0000	125.0	15.3	CNAG_06240	Protein disulfide-isomerase
XM_012191759.1	−3.02	0.0000	127.4	15.8	CNAG_03618	NADPH2:quinone reductase
XM_012194692.1	−3.01	0.0000	216.6	27.0	CNAG_02331	Small subunit ribosomal protein S9
XM_012198481.1	−3.01	0.0000	27.4	3.4	CNAG_05597	Transcription initiation factor TFIID subunit 9/adenylate kinase
XM_012194813.1	−3.00	0.0000	5.5	0.7	CNAG_02520	DNA-directed RNA polymerase III subunit RPC4

### Gal-3 disrupted *C*. *neoformans* extracellular vesicles

**Fig. 7:**
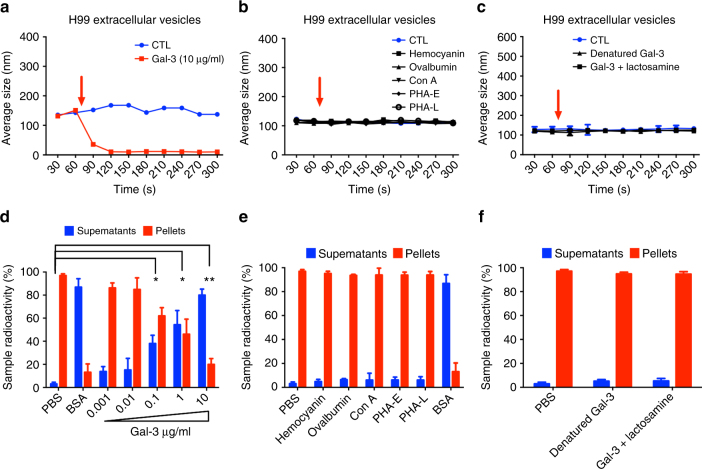
Gal-3 disrupts *C*. *neoformans* extracellular vesicles. Temporal kinetics of vesicle disruption mediated by Gal-3 were measured by dynamic light scattering over ten 30 s to read the population average size (**a**−**c**). The addition of Gal-3 10 μg initially leads to disruption (**a**). The same samples were read first without (blue line) and then with the presence of Gal-3 (red line), which was added between the 60 and 90 s intervals (red arrow). Hemocyanin, ovalbumin, concanavalin A (Con A), phytohaemagglutinin E (PHA-E), and phytohaemagglutinin L (PHA-L) were used as control (**b**). Also denatured Gal-3 and Gal-3 pre-incubated with lactosamine were used as control (**c**). Purified radiolabeled vesicles after 72 h post [1-^14^C] palmitic acid addition were resuspended in PBS, BSA, Gal-3 (0.001 to 10 μg/ml) (**d**), hemocyanin, ovalbumin, Con A, PHA-E, and PHA-L (**e**), denatured Gal-3 and Gal-3 pre-incubated with lactosamine (**f**), and supernatant (blue bars) and pellet (red bars) radioactivity were assessed and normalized to 100% radioactivity for each individual sample. Bars represent the mean ± SD from triplicate samples. Statistically significant differences are denoted by asterisks (*p < 0.05, **p < 0.005, unpaired Student’s *t*-test)

Exposure of fungal EVs to macrophages or bovine serum albumin (BSA) causes vesicular disruption^16^. On the basis that EVs contain capsular components^14^ and that Gal-3 interacted with the capsule of *C*. *neoformans*, we asked whether Gal-3 would affect the physicochemical properties of EVs. Addition of Gal-3 to EVs samples resulted in the shift of a vesicular average size from 150 nm to below the detection limit (approximately 30 nm) within 90 s (Fig. 7a). No other lectins caused similar effects (Fig. 7b). We then evaluated the involvement of the Gal-3 CRD in the putative vesicular disruption. Samples of Gal-3 that were boiled or pre-incubated with its glycoligand (*N*-acetyl-lactosamine) had no lytic effect on *C*. *neoformans* EVs (Fig. 7c), indicating that the Gal-3 CRD is crucial for the Gal-3 lysing activity. Similar results were obtained from assays using radiolabeled vesicles. Gal-3 promoted vesicular disruption and consequent radioactive release in a dose-dependent fashion (Fig. 7d). Radioactive assays confirmed that non-related lectins were unable to lyse *C*. *neoformans* EVs (Fig. 7e). The radioactive assay also confirmed that denaturation or pre-incubation with lactosamine inhibited the Gal-3-induced EV (Fig. 7f).

### Gal-3 impacts the ability of macrophages to disrupt and internalize EVs

**Fig. 8:**
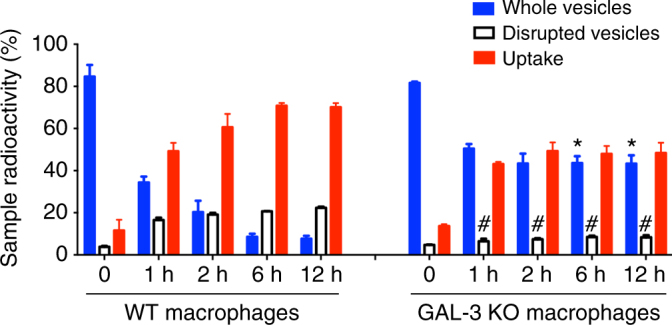
Gal-3 is important for disruption and internalization of *C*. *neoformans* EVs by macrophages. Purified radiolabeled EVs were added to cultures of C57BL6 WT or Gal-3^−/−^ macrophages. After 1, 2, 6, or 12 h post EVs addition, the radioactivity recovered from the macrophages (adhered cells, uptake, red bars), intact vesicles (pellet, blue bars), and disrupted vesicles (supernatant, white bars) were counted by scintillation

Gal-3 is expressed and functional in most macrophage populations^28–30^. Since macrophages^16^ and Gal-3 (this study) can disrupt EVs, we asked whether these events would be correlated. Treatment of macrophages with radiolabeled vesicles suggested that WT phagocytes were approximately three times more efficient than Gal-3^−/−^ cells to disrupt EVs. In addition, we verified that the uptake of radioactive signal by WT peritoneal macrophages have progressively augmented, whereas the uptake by Gal-3^−/−^ macrophages was permanently lower (Fig. 8).

## Discussion

In the current study, we report for the first time the diverse roles played by Gal-3 in *C*. *neoformans* infection. Virtually all bacterial and eukaryotic cells display surface glycans that may be targeted by host carbohydrate-binding proteins. The established interactions frequently influence the microorganism pathogenesis, the host immune response or the occurrence of intracellular parasitism^31^. Galectin-3, a member of the galectin family of beta-galactosides-binding proteins is known by its ubiquitous occurrence throughout the animal species, large distribution in mammals’ tissues, and wide functional diversity^32^.

Activated macrophages express large amounts of Gal-3, which predominates in the cytoplasm^33^. The nucleus, the cell surface, and the extracellular environment are additional sites where Gal-3 can be detected. This distribution is compatible with the multifunctionality of Gal-3 (reviewed by ref. ^33^). Relevant roles are attributed to Gal-3 interactions established during infections (reviewed by ref. ^31^). The successive studies made clear that Gal-3 binding to microbial glycans can positively or negatively regulate pathogen attachment, invasion, and survival, as well as host responses that mitigate microbial pathogenesis^20^.

Knowledge on the functions of Gal-3 in fungal infections is still preliminary. In experimental models of paracoccidioidomycosis and histoplasmosis, Gal-3 influenced the host response by regulating cytokine production^23, 25^. In paracoccidioidomycosis, Gal-3 favored the development of detrimental Th2 immune response, associated with severe disease^23^. Gal-3 was also detrimental to the host resistance against histoplasmosis by negatively regulating IL17-A responses, which was attributed to inhibition of the IL-23/IL17 axis in dendritic cells^25^. These data are consistent with the in vitro demonstration that Gal-3 modulates Th17 response by regulating dendritic cells cytokines^34^. Since Th17 responses are known to be critical in conferring protection against fungal infection, the herein mentioned studies support the notion that Gal-3 plays a negative role in the induction of anti-fungal immunity. This notion was not supported by our current data showing augmented contents of IL-17, IL-23, IL-12p40, and IL-6, in organs of Gal-3-proficient mice infected with *C*. *neoformans*. Our current work suggests that Gal-3 may favor the occurrence of a skewed Th17 immune response. Since we did not detect any increase of IFN-γ, we attribute the elevated detection of IL-12p40 to increased production of IL-23, since this cytokine shares the p40 subunit of IL-12. The occurrence of Th17 immunity in the Gal-3-proficient mice infected with *C*. *neoformans* was reinforced by the augmented detection of IL-6, which is an inductive signal for the IL-17 production by Th17 cells. Moreover, the IL-17 level detected in infected lungs is consistent with that reported by other studies^35, 36^. In fact, IL-17 and Th17 responses are important for the clearance of pulmonary cryptococcosis^35–38^.

The studies performed in models of experimental candidiasis have afforded the most important information available on the roles played by Gal-3 in fungal infections. These showed Gal-3 participation^24, 34, 39^ in the generation of Th17 protective immunity against candidiasis (reviewed by ref. ^40^), a phenomenon verified in this study for *C*. *neoformans*. The studies on experimental candidiasis revealed also that interaction with Gal-3 is responsible for several macrophages responses previously reported as stimulated by β-1,2-linked oligomannosides of *C. albicans* ^41^. The identification of Gal-3 as macrophage receptor for β-1,2-linked oligomannosides was surprising, since members of the galectin family are defined by a common carbohydrate-recognition domain with affinity for β-galactosides^42^. Nonetheless the Gal-3 binding to β-1,2 oligomannosides was largely certified and found triggering macrophages relevant responses, such as increased TNF-α production and fungicidal effect, which account for the death of *Candida* species^43^. Consistently, *C*. *albicans* infection of Gal-3^+/+^ and Gal-3^−/−^ mice showed an association of the deficiency of Gal-3 to the increased susceptibility to the fungal disease^24^; although involvement of Th17 immune response was not explored in their model. We also verified that Gal-3^−/−^ mice are more susceptible *C*. *neoformans* infection, and demonstrated that only Gal-3^+/+^ mice exhibited increased tissue content of IL-17/IL-23 cytokines. Considering the studies on candidiasis, we hypothesized that Gal-3 binding to the *C*. *neoformans* capsule could occur through the recognition of β-1,3 mannosides. However, the known structures of capsular GXM and GalXM contain only β-1,2 mannosides, which are supposedly not recognized by Gal-3^44, 45^. Furthermore, the occurrence of oligosaccharides with terminal β-galactoside, which is the most common ligand of Gal-3, was not verified in *C*. *neoformans*. Therefore, further investment on the identification of the glycoconjugate targeted by Gal-3 is necessary to clarify this important issue.

The finding that Gal-3 had a fungistatic effect for *C*. *neoformans* suggests that this molecule has a role in antifungal defense. To understand how Gal-3 could be mediating its effects, we analyzed *C*. *neoformans* gene expression in its presence and absence. We observed upregulation of genes related to membrane proteins, mainly those involved with sugar transport. Downregulated genes mostly included those related ribosomal activities, suggesting ablation of the functions housekeeping energy-related molecules. Gal-3 caused disruption of *C*. *neoformans* EVs, as well as an efficient uptake of EV contents by macrophages. Identification of the target of Gal-3 in EVs was beyond the scope of the current study, but GXM and GalXM are natural candidates. Serum albumin, a protein used as a positive control in our assays, was previously reported as a vesicle-destabilizing factor in tissue culture medium^16^. The mechanism of disruption remains undefined, but albumin binds fatty acids^46^ and sterols^47^ and likely induces membrane destabilization. Gal-3-induced vesicle lysis, however, might be a novel immunological mechanism because it defeats the putative role of vesicles in delivering a concentrated punch of virulence factors and instead could result in the release and dilution of fungal components into host cells and tissues. The transcriptional changes observed with Gal-3 *C*. *neoformans* imply cellular stress, which combined with the observation that this protein disrupts vesicles, leads to the suggestion that its mechanism of action may be membrane damage that is sufficient to inhibit replication of fungal cells. Thus, increased Gal-3 levels during infection could protect the host by interfering with vesicle-mediated delivery of components that promote *C*. *neoformans* virulence and inhibiting fungal growth.

Taken together, our findings suggest multiple roles for Gal-3 in cryptococcal infection. Gal-3 functions in host defense against *C*. *neoformans* more by interfering with fungal physiology through delayed replication, capsular binding, and vesicle disruption than by its effects on the inflammatory response. In this regard, the role of Gal-3 in host protection against *C*. *neoformans* appears to differ for the effects described with other mycoses. Galectins are known to have direct antimicrobial effects on variety of bacteria and are fungicidal to *C*. *albicans* ^43^. Hence, Gal-3 appears to be part of innate immunity against *C*. *neoformans*. These results provide a new window on our understanding of the immunopathogenesis of cryptococcosis and suggest a novel target for the design of therapeutic agents to combat this mortal mycosis.

## Methods

### Ethics statement

All animal use complied with the standards described in Ethical Principles Guide for the Care and Use of Laboratory Animals adopted by the Brazilian College of Animal Experimentation and Albert Einstein College of Medicine Institutional Animal Care and Use Committee guidelines. The protocols were approved by the Committee of Ethics in Animal Research of the Ribeirao Preto Medical School at the University of Sao Paulo (protocol 100/2015). Informed written consent from all participants was obtained. The studies involving patients were approved by the Research Ethics Committee of the University Hospital, Ribeirao Preto Medical School at the University of Sao Paulo (protocol HCRP 4.096/2012).

### Mice and *C*. *neoformans* isolates

We used male C57BL/6 (wild-type, WT, Jax 000664) and Gal-3-deficient mice (gal3^−/−^) at 6−8 weeks of age. Knockout mice were kindly donated by F.T. Liu (University of California, Davis, CA). Gal3^−/−^ mice were previously generated as described and bred on the C57BL/6 mouse background for nine generations^48^. The animals were housed in the animal facility of the Ribeirao Preto Medical School, University of São Paulo, under optimized hygienic conditions. All *C*. *neoformans* experiments were conducted with the clinical isolate H99 strain (serotype A) or *C*. *neoformans* acapsular strain (CAP67). The *C*. *neoformans* H99 strain was generously provided by John Perfect (Durham, NC) and the CAP67 strain was purchased from the American Type Culture Collection (Manassas, VA). Fungal cultures were grown in minimal medium composed of dextrose (15 mM), MgSO_4_ (10 mM), KH_2_PO_4_ (29.4 mM), glycine (13 mM), and thiamine-HCl (3 M). Fungal cells were cultivated at room temperature (RT) with continuous shaking (150 rpm). To maintain fungal virulence, serial passages in C57BL/6 mice were performed before the isolate H99 was used in experiments.

### *C*. *neoformans* infection and survival analysis

The H99 strain was grown in minimal medium until late logarithmic phase, washed and suspended in PBS. The concentration of yeast cells was determined by hemocytometer, and a suspension of 1×10^6^ cells/ml was used to infect the mice via intratracheal with 50 μl of solution. The WT and gal3^−/−^ mice were anesthetized with xylazine (125 mg/kg) and ketamine (10 mg/kg) in PBS, and their necks were hyperextended and their trachea was exposed after incision. The infection with *C*. *neoformans* used a syringe 26-gauge, and the incision was sutured with 5–0 silk. The inocula of 5×10^5^ cells were realized to measure the fungal burden, the levels of cytokines, or the relative expression of transcription factors in the brain, lung, spleen, and serum. These measurements were evaluated on days 3, 7, and 14 days after infection. The infection with 1×10^5^ yeast cells was performed to survival analysis of WT and gal3^−/−^ mice, which were monitored daily for mortality. The concentration of inocula was confirmed by plating on Sabouraud Dextrose Agar (Difco, Detroit, MI, USA) and counting the CFU.

### Organ fungal burden

The *C*. *neoformans* burden was quantified in brain and lung homogenates. Sample of homogenate was diluted in sterile PBS buffer (pH 7.2) and aliquots of 100 μl were plated in Sabouraud agar medium. After 48 h of incubation at 30 °C, the CFU were counted and calculated the concentration of yeast cells/ml. These values were related to organ mass and the *C*. *neoformans* burden for each organ was expressed as CFU/ml/mg of tissue.

### Sera and patients

Blood samples were obtained from patients being seen in the University Hospital, Ribeirao Preto Medical School at the University of Sao Paulo. A total of six patients with diagnosed cryptococcosis were included in this study: three patients with HIV and three patients IC. Serum was obtained and stored at −80 °C. Samples were also obtained from three blood donors with a median age of 30 years (range, 25–35 years).

### Gal-3 levels

The Gal-3 levels in the brain, lung, and spleen was quantified in the organ homogenates of *C*. *neoformans*-infected mice. The homogenate samples (whole organ in 1 ml of PBS) of control mice or from animals infected with *C*. *neoformans*, as well as the serum samples from infected animals or from patients with diagnosed cryptococcosis, were stored at −80 °C until assayed. All samples were thawed only once prior to use. Gal-3 levels were measured using commercially available enzyme-linked immunosorbent assay (ELISA) kits (Sigma-Aldrich, St. Louis, MO, USA) according to the manufacturer’s instructions.

### Growth in the presence of Gal-3

*Cryptococcus neoformans* strains H99 (capsular) and CAP67 (acapsular) cells were grown in Sabouraud medium at 37 °C with 150 rpm. To verify the Gal-3 effect on the cells, we performed growth curves in Sabouraud liquid medium containing different concentrations of Gal-3 (Gal-3 human recombinant, expressed in *E*. *coli*, Sigma-Aldrich) in a 96-well plate (Costar, NY, USA) using an iEMS spectrophotometer (Thermofisher). The optical density at 540 nm (OD540) was measured every hour, and graphs plotted using *GraphPad Prism* Software version 6.0 (GraphPad Software, San Diego, CA, USA).

To measure the capsule and body size of the cells, we analyzed H99 strain after 72 h of Gal-3 treatment and compared to the cells treated with PBS. Suspensions of India ink were photographed and measured. Image acquisition was done at differential inference contrast objective in a SP5 confocal inverted microscope equipped with a camera. Images were processed using Leica Microsystems and ImageJ software. Capsule and cell body dimensions were determined using Adobe Photoshop. Capsule radial length was calculated by subtracting the length of the cell body from the diameter of the whole cell, capsule included.

### Gal-3 binding assay

The *C*. *neoformans* strains H99 (capsular) and CAP67 (acapsular) cells were incubated with PBS/10% bovine fetal serum for 20 min at 4 °C to prevent nonspecific antibody binding. After washing, H99 and CAP67 cells at a density suspension of 1×10^6^ cells/ml were incubated with native and denatured form of Gal-3 (40 μg/ml) for 40 min at 4 °C. The cells were washed twice with PBS and the anti-Gal-3 antibody (1:50; Sigma-Aldrich) was added; after 45 min of incubation, the cells were washed with PBS and incubated with anti-rabbit IgG-FITC antibody (1:50; Sigma-Aldrich) for 40 min at 4 °C. Gal-3 binding to H99 and CAP67 cells was analyzed by flow cytometry (Guava easyCyte, Guava Technologies, Millipore, Hayward, CA, USA) and the histogram represents the percentage of positive cells recognized by Gal-3. The anti-rabbit IgG-FITC antibody associated or not with Gal-3 was used as control, and use of WGA lectin (30 μg/ml) as control of binding with capsule or cell wall.

### Confocal microscopy

*C*. *neoformans* yeast cells (H99 strain) were cultured at 37 °C for 24 h. The cells were incubated with Gal-3 (40 μg/ml) at 37 °C for 1 h, followed by washing with PBS and fixation with PBS-buffered 3.7% formaldehyde at 25 °C for 1 h. After fixation, cells were washed with PBS and embedded in OCT-Tissue Tek (Electron Microscopy Sciences, Hatfield, PA, USA). The samples were frozen and processed as described previously^49^. Slides were rinsed three times with PBS before and after treatment with glycine (0.1 M, 15 min), and blocked with BSA (1% in PBS) for 1 h. Samples were incubated overnight at 4 °C with murine monoclonal antibody 18B7 (IgG1, 1 μg/ml), which binds cryptococcal glucuronoxylomannan (GXM) from all serotypes^50^. After washing with PBS, the cells were incubated with a rabbit anti-Gal-3 antibody (Sigma-Aldrich) overnight at 4 °C. The samples were washed five times with PBS and incubated for 1 h with or FITC-labeled secondary antibodies, which included Alexa Fluor 594-labeled goat anti-mouse IgG from Thermo Fisher Scientific and a FITC-labeled donkey anti-rabbit IgG from Jackson Immuno Research Laboratories. For cell wall detection, samples were incubated with Calcofluor White (50 μg/ml) (Sigma-Aldrich) in PBS for 20 min. After five washes with PBS (5 min each), coverslips were mounted with Fluoromount-G (Electron Microscopy Sciences). Samples were treated with pre-immune serum from mice or rabbit and later treated with secondary antibodies as nonspecific labeling control. The samples were examined with a LSM780 system AxioObserver, 63 X oil immersion (Carl Zeiss, Jena, Germany). The images were analyzed offline using the ImageJ software (http://rsb.info.nih.gov/ij/).

### Cytokine measurements in tissues

The homogenates of lung, brain, and spleen were centrifuged at 2000×*g* for 15 min at 4 °C. The supernatants were used to quantify the levels of IFN-γ, IL-12p40, IL-6, TNF-α, and IL-10. The cytokines were measured by capture ELISA with antibody pairs purchased from BD Biosciences (Pharmingen, San Diego, CA, USA). The ELISA was performed according to the manufacturer’s protocol. The cytokine concentrations were determined from standard curves, using murine recombinant cytokines as a standard. The absorbance was read at 450 nm in a microplate scanning spectrophotometer (Power Wave-X; BioTek Instruments, Inc., Winooski, VT, USA). The cytokine concentrations were determined in ρg/ml and related to organ mass, then expressed in pg/ml/mg.

### Quantitative reverse transcription (qRT)-PCR of transcription factors

Total RNA was obtained from lungs by TRIzol Reagent, according to the manufacturer’s instructions. Reverse transcription of RNA into cDNA was done by the ImProm-II Reverse Transcription System (Promega, Fitchburg, WI, USA) using oligo (dT). The qRT-PCR was performed in 15 μl reactions with SsoFast^TM^ EvaGreen (Bio-Rad Laboratories, Hercules, CA, USA). The reactions were read in the Bio-Rad CFX96 Real-Time PCR System (Bio-Rad Laboratories) using the following conditions: 95 °C for 30 s, and 40 cycles of 95 °C for 5 s/60 °C for 5 s. Gene expression was quantified using the ΔΔCt method and normalized to β-actin expression. The utilized PCR primers were: β-actin (F: 5′-AGCTGCGTTTTACACCCTTT-3′ / R: 5′-AAGCCATGCCAATGTTGTCT-3′); T-bet (F: CACTAAGCAAGGACGGCGAA / R: CCACCAAGACCACATCCAC); GATA-3 (F: AAGAAAGGCA TGAAGGACGC / R: GTGTGCCCA TTTGGACA TCA); ROR-γ t (F: TGGAAGATGTGGACTTCGTT / R: TGGTTCCCCAAGTTCAGGAT).

### Flow cytometric analysis of pulmonary leukocytes

Pulmonary leukocytes were isolated as described previously^51^ with slight modifications. WT and gal3^−/−^ mice infected with *C*. *neoformans* (H99 strain) were killed on day 3 post-infection and their lungs removed aseptically. The lungs were fragmented and digested with type II collagenase (1 mg/ml; Thermo Fisher Scientific) at 37 °C for 30 min. The cells were filtered on nylon filters (70 μm; Thermo Fisher Scientific) and washed with sterile PBS. For erythrocyte elimination, cell suspensions were treated with 500 μl of NH_4_Cl buffer for 5 min on ice followed by the addition of 10-fold excess PBS. Leukocytes were centrifuged at 800×*g* for 5 min, washed twice with sterile PBS and suspended in sterile PBS containing 2% heat-inactivated fetal bovine serum (FACS buffer). The cells were counted in a Neubauer’s hematocytometer and adjusted to the density of 10^7^ cells/ml. Fifty microlitres of Fc block monoclonal antibody was added to each 100 µl cell suspension, followed by incubation for 30 min on ice to inhibit non-specific binding. The cells were then incubated with a biotinylated hamster anti-mouse γδ T-cell receptor antibody (0.5 mg/ml, BD Biosciences) for 1 h on ice. After washing with PBS, the cells were incubated with FITC-conjugated streptavidin (10 μg/ml, Thermo Fisher Scientific). The frequency of γδ T lymphocytes present in the pulmonary leukocytes was obtained by flow cytometry using a BD FACSCalibur (BD Biosciences) flow cytometer.

### Analysis of the stability of extracellular vesicles

EVs were obtained as previously described^52^, with slight modifications. After fungal cells growth until the stationary phase (1×10^7^ to 10^8^ cells/ml), culture supernatants were separated by sequential centrifugation at 4000×*g* and 15,000×*g*. Pellets were discarded, and the supernatants concentrated through Amicon ultrafiltration system with a 100-kDa cutoff (Millipore, Billerica, MA, USA). The obtained material was ultracentrifuged at 100,000×*g* for 1 h at 4 °C. The supernatants were discarded, and the pellets were washed three times with PBS, by using successive steps of resuspension and centrifugation, at 100,000×*g* for 1 h at 4 °C. Vesicles detection was based on the sterol presence in their membranes by using a quantitative fluorimetric Amplex Red sterol assay kit (Molecular Probes, Thermo Fisher Scientific), according to the manufacturer’s instructions. The EVs stability was evaluated according to protocols previously described^16^, with slight modifications. Briefly, EVs incubated at 37 °C, 5% CO_2_, for 1 h, with Gal-3 (#G5170, Gal-3 human recombinant, expressed in *E*. *coli*, Sigma-Aldrich) at different final concentrations (0−10 µg/ml). The concentrations of all control lectins were normalized according to carbohydrate binding sites. Vesicle size was estimated by dynamic light scattering, for each experimental condition. Data were expressed as the average of three runs. EVs stability was also examined through a radioactive assay through cultivation of *C*. *neoformans* in the presence of [1-^14^C] palmitic acid^16^. The suspension of radiolabeled EVs was incubated with Gal-3 under the conditions described above and the suspension was ultracentrifuged at 100,000×*g* for 1 h at 4 °C. Supernatants and pellets were saved for scintillation counting.

### Vesicle disruption and uptake by macrophages

To study vesicle stability and vesicle uptake by macrophages from WT and gal3^−/−^ mice, we used a protocol that was previously described^16^, with slight modifications. Thioglycolate-elicited macrophages were harvested from the peritoneal cavity of C57BL/6 WT or gal3^−/−^ mice, and grown in DME medium (Invitrogen) supplemented with 10% (v/v) fetal bovine serum, 10% NCTC (Invitrogen), 1% nonessential amino acids (Invitrogen) and 1% penicillin (Invitrogen). Forty-eight-well tissue culture plates were seeded with elicited peritoneal macrophages (4×10^5^ cells/well). EVs were obtained from *C*. *neoformans* cultures that were pulsed with [1-^14^C] palmitic acid 72 h before EVs harvesting, as previously described^16^. A volume of 200 μl of vesicle-containing medium was added to each well. Controls included vesicle-containing medium alone. After 1, 2, 6, or 12 h incubation at 37 °C in 9.5% CO_2_, culture supernatants were collected. The adhered cells were also collected, washed three times with PBS, lysed in 200 μl of 25 mM deoxycholate and the resultant material was collected for scintillation counting. The harvested material from the culture supernatants was ultracentrifuged at 100,000×*g* for 1 h at 4 °C. Supernatants (containing components of disrupted EVs) and pellets (containing intact EVs) were saved for scintillation counting. The radioactivity distribution in the three fractions was expressed as percent of the total radioactivity.

### RNA sequencing and data analysis

Total RNA was obtained from three biological replicates of *C*. *neoformans* cells (H99 strain) incubated with minimal medium or Gal-3 (0.1 μg/ml in minimal medium) for 24 h at 37 °C. The cells were harvested by centrifugation, frozen in liquid nitrogen, and lysed with the use of mortar and pestle for total RNA isolation. Total RNA was obtained using Trizol (Invitrogen), treated with RNase-free DNA I (Fermentas), and cleaned up using an RNAeasy Kit (Qiagen Biotecnologia Brasil Ltda, Sao Paulo, SP, Brazil) following the manufacturer’s instructions. The RNA was quantified using a Qubit 3.0 Fluorometer (Life Technologies), and analyzed using an Agilent 2100 Bioanalyzer system to assess the RNA integrity, which ranged from 9.5 to 9.9 (integrity number). The barcoded libraries were prepared and sequenced in the Center for Medical Genomics of the Ribeirao Preto Medical School (Ribeirao Preto, Brazil) using the Illumina Truseq mRNA Sample Preparation kit according to the manufacturer's recommendations. Subsequent clustering and sequencing were performed with the Illumina NextSeq 500 system. Paired-end reads were de-multiplexed and analyzed against *C*. *neoformans* reference genes using the Kallisto software (version 0.43.1)^53^, resulting in an average value of 21M reads per sample. Differentially expressed genes were analyzed using edgeR^54^. Gene expression changes in the range of >1.0 or <−1.0 log fold (*p* < 0.05) were classified as biologically and statistically significant.

### Statistical analysis

Data are either the means of or representative results from at least three independent experiments, each performed in triplicate. All statistical analyses and comparisons were performed using the *GraphPad Prism* Software version 6.0 (GraphPad Software, San Diego, CA, USA). The log rank test was used to compare the survival rates between the study groups. A *p*-value < 0.05 was considered statistically significant.

### Data availability

The RNA sequence data have been deposited in NCBI under BioProject accession number PRJNA415606. All other relevant data supporting the findings of the study are available in this article and its Supplementary Information files, or from the corresponding author upon request.

## Electronic supplementary material

Supplementary Information

Description of Additional Supplementary Files

Supplementary Data 1

Supplementary Data 2

Supplementary Data 3

Supplementary Data 4
