# Supplementary material for: Galectin-3 impacts Cryptococcus neoformans infection through direct antifungal effects
Source: Nat Commun. 2017 Dec 6;8:1968. doi: 10.1038/s41467-017-02126-7 (PMC5719036; doi:10.1038/s41467-017-02126-7)
Supplement: Supplementary file 1 — Supplementary Information [file 41467_2017_2126_MOESM1_ESM.pdf]

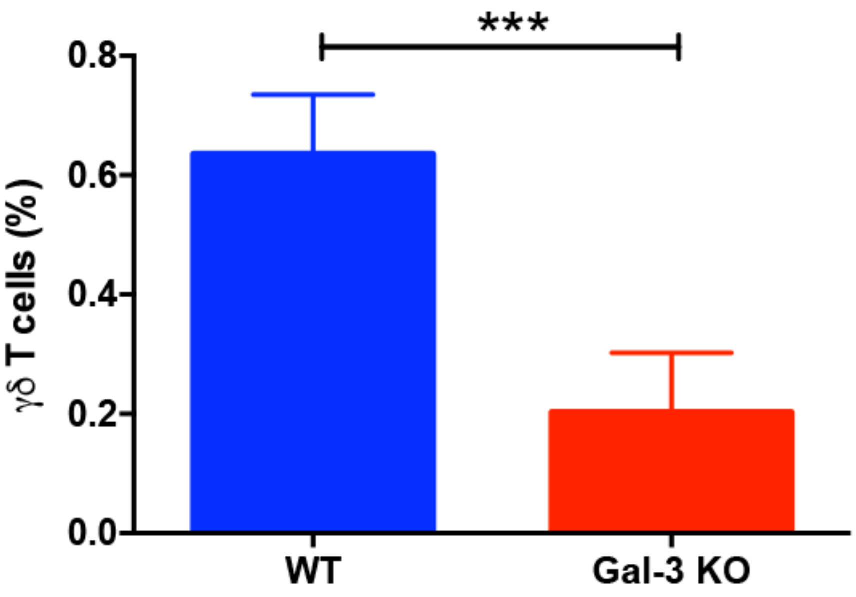

Supplementary Figure 1. Evaluation of γ/δ T cells population in WT and Gal-3 KO mice during experimental pulmonary cryptococcosis. WT (blue bar) and Gal-3 KO (red bar) mice infected with *C. neoformans* were sacrificed on day 3 post-infection and leukocytes were probed for the presence of the γ/δ T-cell receptor. The bars represent means ± standard deviations. ****P*<0.001, unpaired Student's t-test, 5 mice per group. Data are representative of three experiments.

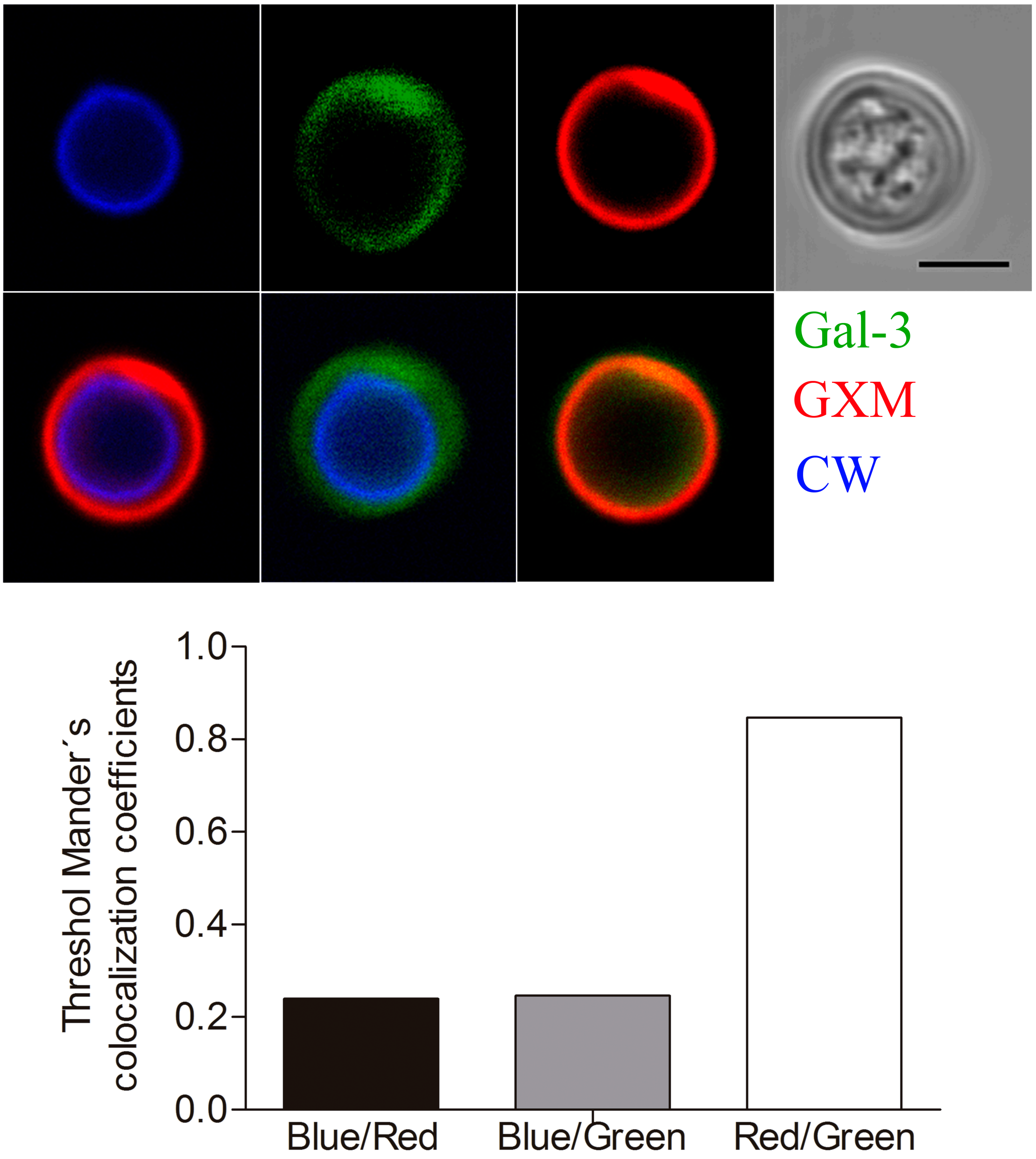

Supplementary Figure 2. Cellular binding sites of Gal-3 in *C. neoformans*. Cellular structures analyzed by fluorescence microscopy included cell wall (calcofluor white, CW, blue fluorescence; A), Gal-3 binding sites (anti-Gal-3 antibody, green fluorescence; B), and capsule (18B7 antibody, GXM, red fluorescence; C). Merged images are shown in E (capsule and cell wall), F (Gal-3 and cell wall) and G (Gal-3 and capsule). *C. neoformans* is also illustrated under DIC (D). The images represent a single section from a Z series stack. Scale bar = 3 µm. Co-localization of Gal-3 binding sites with capsular structures was confirmed by the

threshold Mander´s coefficient tool available in the Fiji J software (H). The whole image field was used to obtain the split threshold Mander´s colocalization coefficient for each channel (Tm1/Tm2).
