# Supplementary material for: Galectin-3 impacts Cryptococcus neoformans infection through direct antifungal effects
Source: Nat Commun. 2017 Dec 6;8:1968. doi: 10.1038/s41467-017-02126-7 (PMC5719036; doi:10.1038/s41467-017-02126-7)
Supplement: Supplementary file 2 — Description of Additional Supplementary Files [file 41467_2017_2126_MOESM2_ESM.pdf]

Description of Additional Supplementary Files

File Name: Supplementary Data 1
Description: Up regulated genes with annotated functions in C. neoformans upon exposure to Gal-3.

File Name: Supplementary Data 2
Description: Down regulated genes with annotated functions in C. neoformans upon exposure to Gal-3.

File Name: Supplementary Data 3
Description: Up regulated genes with hypothetical functions in C. neoformans upon exposure to Gal-3.

File Name: Supplementary Data 4
Description: Down regulated genes with hypothetical functions in C. neoformans upon exposure to Gal-3.
